# Professor Bartlett

**Published:** 1844-06

**Authors:** 


PROFESSOR BARTLETT.

This accomplished and popular teacher has resigned his professorship in the Transylvania medical school, and accepted of a chair in the University of Maryland. Professor Bartlett's learning, talents and address rendered him an ornament to the institution which he has left, and qualify him for great usefulness in the new sphere to which he is about to remove. He carries with him the respect and good wishes of all his brethren in the West. Success crown his efforts to make the ancient school of Baltimore one of the first in America! He is succeeded by Dr. L. G. Watson, of N. C. Y.

